# The Bright Side of Social Media: Social Media Platforms Adoption and Start-Up Sustainability

**DOI:** 10.3389/fpsyg.2021.661649

**Published:** 2021-06-07

**Authors:** Muhammad Saeed Mujahid, Muhammad Shujaat Mubarik

**Affiliations:** ^1^Department of Marketing and Entrepreneurship, Mohammad Ali Jinnah University, Karachi, Pakistan; ^2^College of Business Management (CBM), Institute of Business Management, Karachi, Pakistan

**Keywords:** start-up sustainability, social media adoption, social media networks, start-up ecosystem, social media platform

## Abstract

This study aims to explicate the contribution of social media platforms adoption on start-up sustainability. Since most economies of the world start-up failure rate are relatively high, there is always a desire or a need to investigate the success recipe. As a result, the primary objective of this study is to understand the social media environment and how start-ups can best utilize social media platforms throughout their life cycle. Based on the qualitative case study approach, five in-depth interviews of social media marketers and individuals working in start-ups were conducted. The finding demonstrates that social media is a crucial virtual platform for striving resource start-ups. Therefore, if a platform gets utilized correctly, it can play an essential role in the sustainable progression of a start-up. Thus, there is a need for start-ups to articulate a comprehensive social media policy for each life cycle stage.

## Introduction

The world has entered the twenty-first century in possession of disruptive technologies, such as the Internet, cell phones, artificial intelligence, robotics, and renewable energy, to name a few. These technologies have paved the way for the creation of disruptive innovation that brings a different set of value propositions in the market (Yovanof and Hazapis, 2008). The study has further added that disruptive innovations redefine the marketplace by creating a value network, thereby disrupting existing market players, products, and alliances. Thus, these technologies are accelerating the pace of product innovation manifold and have significantly reduced the product life cycle (Mujahid et al., 2019). Hence, there is a remarkable effect on the rate of innovation and business development. Therefore, countries such as Finland, Germany, and Sweden, which have supported science, research, innovation, and sustainable development as a national priority, are now among the most prosperous nations (Petru et al., 2019). These nations have environmental sustainability, infrastructure, equity, and an equal chance for each member of the society to succeed. Thus, it has created a need for dynamic organizations that can rapidly carry out research and development (R&D) and continuously bring new products and services to the market. As a result, start-ups get created worldwide in the millions, because they are considered the source of rapid product development. Start-ups have significant contributions to job creation and growth at national, international, and industrial levels (Chodokufa, 2009; Andzelic et al., 2011); thus, they have significantly contributed to economic development (Martínez-Fierro et al., 2020). Start-ups have led to numerous innovative breakthroughs resulting in entirely new businesses (Hathaway, 2016). Therefore, start-ups are believed to be a dynamic enterprise created upon a creative idea for the socio-economic development of a nation. Proper criteria to define a start-up scope are new, active, and independent (Petru et al., 2019). However, many have emphasized innovativeness (Gimenez-Fernandez and Beukel, 2017). Thus, the ultimate goal of a start-up is new product placement, developing information technology, and innovating a process in a global environment while aiming at a long-term gain (Stolze et al., 2014; Frederiksen and Brem, 2017; Mercandetti et al., 2017). To achieve this, technology-enabled start-ups, such as Airbnb, Uber, and other start-ups have innovatively utilized existing technologies to have a significant advantage over their competitors by improving the efficiency of existing products or services. On the other hand, there are high-tech start-ups such as Google, Tesla, and few others who have created technological innovation in their respective fields.

According to Blank and Dorf (2012), vision alone is not enough for start-up sustainability; and there are nine prominent elements: having a target audience, value proposition, customer relationship for demand creation, channels of distribution, vital resources, including suppliers and commodities. Besides crucial partners with other ecosystem members, cost structure, revenue streams, and tasks a start-up essentially performs to attain sustainability contribute to start-up development. In this context, social media is playing a crucial role in the sustainable growth of the start-up (Cestyakara and Surendro, 2013; Gloor et al., 2020). Start-ups are resource-scarce enterprises (Moogk, 2012). Thus, right from the start of their journey, social media is an effective and efficient tool to directly reach out to various players in a start-up ecosystem (Cestyakara and Surendro, 2013; Gloor et al., 2020). Moreover, it enables enterprises to engage customers in mutually beneficial and delightful user experiences (Khan et al., 2020). However, scholars believe that if a customer becomes a victim of cyberbullying, it might create a negative online experience for that individual (Cao et al., 2019).

Moreover, social media tools provide a new way of communication and information sharing among the various players in a start-up ecosystem (Mohout and Fiegenbaum, 2015; Park et al., 2017). Evolving technologies are gaining recognition because they play a key role in creating networking business enterprise networks (Liu and Liu, 2009; Bell and Loane, 2010). Evidence has shown that business enterprises taking advantage of social media technologies have outperformed competitors, and have enhanced efficiencies and reduced cost (Harris and Rea, 2009). Thus, it is vital to understand the impact of social media on the performance of a firm (Wetzstein et al., 2011).

Start-ups, at the onset of their journey, have to demonstrate that they are superior to their peers by the technology they have opted for and the market potential of their offering (Marcus et al., 2013). At its inception, start-ups, because of the absence of production and operational history, have to face many challenges (Zheng et al., 2010; Villanueva et al., 2012). That is why past trends reveal that more than 60% of start-ups fail during the early stage of the life cycle (Lai and Lin, 2015; Melegati et al., 2019). This high failure rate in resource-constrained start-ups is due to various factors that include significant competition (Passaro et al., 2020). The intensity and shape of the challenges of start-ups vary with changes in the life cycle (Gómez, 2007). These challenges arise because of lack of managerial and business knowledge, technological lag, human resources and team management issues, and finally, lack of financial resources that start-ups face over their life cycle (Barazandeh et al., 2015). Hence, start-ups have to strive for their existence and struggle for survival during their life cycle (Salamzadeh and Kawamorita Kesim, 2015). Thus, having a brilliant idea is not good enough for the progression of these start-ups. Instead, there is a consensus among researchers that they must build collaborative relationships with the other players in an ecosystem to enhance their probability of survival and sustainability (Shwetzer et al., 2019; Tripathi et al., 2019). These interconnected players in the start-up ecosystem community formally or informally support start-up creation and sustainability (Mason and Brown, 2014; Isenberg and Onyemah, 2016).

Social media has made it easy for start-ups and other businesses to communicate with their customers, suppliers, and facilitating firms. Thus, these technologies impact how start-ups operate, and how they interact within the start-up ecosystem (Park et al., 2017). Although there is extensive use of social media in business firms, little is known about the impact of technology and social media tools on sustainable performance (Denyer et al., 2011). This acts as an impetus for this study to investigate what role social media can play in uplifting the sustainable performance of start-ups at various stages. Fischer and Reuber (2014) stressed the need to study social media: how an entrepreneur should use it so that researchers on new entrepreneurial ventures can more vigorously study the impact of researchers. The study also explores appropriate social media strategies that start-ups could adopt to engage customers and other stakeholders in the start-up ecosystem during various lifecycle stages, mainly to improve brand awareness and engage the audience. Thus, media adoption would help start-ups to achieve sustainability by employing social equity and economic viability. The study adopts a case study approach and contributes to the literature in the following ways. First, the study provides an in-depth understating of the role of social media in various stages of start-ups. Second, this study highlights social media strategies that could be instrumental in increasing the survivability chances for failing start-ups. In short, the study highlights the bright side of social media in uplifting sustainable performance by providing a platform to create networking with ecosystem players and access to resources that were earlier not available to them. Moreover, it would enhance the efficiency of start-ups and the effectiveness that lead to their sustainability.

## Literature Review

### Start-Ups: Evolution and Life Cycle Stages

Scholars have long since adopted the biological notion of the life cycle of an organization researcher (Quinn and Cameron, 1983). They have suggested that the evolution of an organization is through different life cycle stages during its journey from start-up to growth (Mintzberg, 1984; Dodge et al., 1994; Hanks et al., 1994). Startup progression is vibrant, nonlinear, and volatile. Bygrave (1989) has used chaos theory as a symbol for organizational progression under and group cultivation. The study further added that this theory can be employed for studying startups and new product development process. Occurrence of an event results in the startup. Development of startup is dynamic, co-evaluate with management (McKelvey, 2002), startup do not achieve equilibrium point just like we have in biological species.

Generally, start-ups have been associated with technological projects, since most of their solution is backed by software produced and reproduced effortlessly (Klačmer Calopa et al., 2014). Hathaway (2016) has characterized start-ups as a yet-to-be-formed business, built upon an innovative business model, financed by start-up capital. Start-ups usually aim to create an innovative product backed by the latest technology. It can achieve a high growth rate by exploring scalable markets (Paternoster et al., 2014). Zajko (2017) has defined start-ups as an enterprise in the early phase of its life cycle. Literature shows that different scholars studying the lifecycle of a start-up have varying phases of start-up progression. The stages suggested by the researchers have varied from a minimum of three to a maximum of five. However, life cycle model fusion concludes that the generic life cycle of an organization passes through five stages: start-up, expansion, consolidation, diversification, and decline (Hanks, 1990). While the study further adds that scholars differ on the number of stages through which an organization passes over the life cycle, there are commonalities.

Similarly, there is an apparent disagreement on the activities accompanied at each stage. There are similarities and differences in the phases of start-up progression mentioned in the literature. According to Crowne (2002), the first stage is about product development and sale. Further added by the study is that once sales start maturing, the start-up firm reaches the stability stage and now focuses on getting an IPO. Picken (2017) has suggested that working with technology development as an initial stage is followed by a transition period that converts the start-up from an informal organization structure into a structured one. It helps them in the scale-up period, which is the make-or-break stage; and according to the author, a start-up unable to scale will lose its journey and will cease to exist. Whereas, Blank (2013) has suggested two models, a product development commences with vision converting into business planning while ascertaining the related issues, and a customer development model begins the journey by identifying customer problems and coming with its solution.

However, for this study, we have adopted the start-up life cycle model that has been suggested by Maurya (2012). According to the study, the lifecycle of start-ups passes through three stages of development.

#### Problem/solution Fit Stage

A start-up life cycle instigates a problem/solution fit by looking into whether a market has identified any problem requiring a solution. Thus, start-up just needs to solve the market problem. However, the given solution must align with the problem, and the customer is ready to give it a try. In such a scenario, idea generation is not that important.

#### Product/market Fit Stage

During the product/market fit stage, the start-up team looks into whether the market requires the executed idea. Therefore, after verifying the problem in the earlier stage, it is now time to conduct analyses to determine the utility of the new product in light of its issues.

#### Scale Phase

The third phase of a start-up is the scale phase. After successfully testing the market for the suggested solution, it is time to look for growth; it means market expansion, adding new employees. Now start-up will be looking at potential growth.

### Start-Up Sustainability

Start-up is a composition of scalable and replicable business models working in a risky and uncertain environment. Since start-ups are created upon an innovative idea, they grow at a rapid pace. Thus, it requires more than just capital to be a radical innovator. This entails a predisposition to risk toward experimentation, even failure (Shahzad et al., 2020).

Moreover, start-ups use initial years to develop products without sales, so they depend on funding. There are cases in which 50% of funded start-ups have almost zero output (Nanda and Rhodes-Kropf, 2013). Therefore, if they can survive early years and achieve what has been required by founding members, it is an achievement on their part (Silva et al., 2016). There are many ways to measure start-up sustainability depending on entrepreneurship goals, and it can be financial or non-financial founder team performance or pre-defined expectations.

Moreover, empirical studies suggest that start-up endurance, planning, and product excellence are not enough to attain sustainability (Dahlqvist et al., 2000). Instead, it is advocated that looking for the right customers and caring for them is far more critical. Thus, marketing activities have a considerable impact on the sustainability of start-ups. Start-ups with limited financial resources use social media platforms for promotion, customer education, and creation of brand awareness (Hutter et al., 2013; Seo and Park, 2018). It is recommended that start-ups right from the nascent stage focus on creating brand awareness, conveying the competitive advantage of the offering. Since these things are absent in the early years, it is relatively challenging for them to create relationships with their customers, suppliers, creditors, and other organizations for a mutually beneficial exchange. Consequently, it is tough to develop market channels and acquire financial and non-financial resources. Given that customer satisfaction plays a vital role in business sustainability, to achieve this goal, a start-up should ensure the attention of customers through communication campaigns and brand support (Petru et al., 2019). However, it takes time for an enterprise to create goodwill and brand equity (Korunka et al., 2010; Lohrke et al., 2010).

### Social Media and Start-Ups

The inception of the World Wide Web (www), an invention of social media platforms like Twitter, WhatsApp, and LinkedIn, has revolutionized the way individuals communicate and keep in touch with each other, and has drastically changed since then. Simultaneously, individuals can also communicate with their friends and family members to discuss start-up ideas. Social media has become an essential part of our lives (Bano et al., 2019; Khan et al., 2019). Social media is characterized as a cluster of online portals that facilitate its users' in getting audience feedback, share information, encourage participant contribution, allow voting rights, one-to-one communication, and form communities with a common interest (Mayfield, 2008). Kaplan and Haenlein (2010) have described social media as a bunch of Internet-based applications built on the technological and ideological foundations of web 2.0 that allow two-way conversation between start-ups and customers (Fournier and Avery, 2011).

Moreover, previous studies on social media have highlighted the multitude of changes social media has bought to society and its far-reaching effects on how we do business and entrepreneurial ventures work (Fischer and Reuber, 2011, 2014). These technologies have brought about new information and communication tools that have taken the business world and entrepreneurial firms by storm. However, the most talked-about and impactful platform is Facebook, because of the sheer number of users and many features to facilitate communication within social media platforms (Di Capua, 2012).

The increasing power of the Internet and Internet-based social media has challenged the market dynamics and competitiveness of all types and sizes of businesses, stretching from the seasoned corporate sector to newly created start-ups. The rise of social media applications has transformed the start-up ecosystem as well. There is increasing popularity of social media tools in business firms, whether they are start-ups or large corporations (Andriole, 2010; Bell and Loane, 2010). The launching of social media has sent shockwaves in the world of marketing, news media, and even politics (Gruber et al., 2015). This change has provoked marketing firms and businesses to reevaluate their relationship with their stakeholders and reconsider their communication methods (Kietzmann et al., 2011). Thus, social media has transformed into an inevitability for businesses nowadays (AlSharji et al., 2018).

Since the early stage of the life cycle, a start-up has to manage anxiety due to uncertainty in the environment. Moreover, start-ups at the nascent stage have to undertake several sequential activities, such as conducting market research, developing a prototype, hiring new employees, and seeking funds while creating a new venture (Gartner, 1985). To accomplish this, Fischer and Reuber (2011) have suggested that start-ups should use good communication tactics on social media platforms to secure and build an early clientele. In this respect, Facebook is an influential media, since it has many features that facilitate the entrepreneurial ecosystem, such as Facebook groups, calling, and messaging on Facebook messenger. The two more popular social media platforms are Twitter and Facebook (Van Dijck, 2013), becoming an essential resource for young entrepreneurs to connect themselves with the ecosystem. Due to the ineffective nature of communication in the pre-social media era, accessing information, and sharing ideas were challenging for young entrepreneurs (Park et al., 2017). Thus, social media might be used to create business partnerships and collaborations.

Moreover, a social media platform is used for information sharing, thereby creating trust and an enabling environment (Raza et al., 2020). Twitter can be used as a catalyst in this role to enhance business-to-ecosystem and business-to-business interaction for start-ups. Fischer and Reuber (2011) have observed that the word “entrepreneur” is the most common word found in the Twitter biography section of the profile of an individual. Pitafi et al. (2020) have suggested that Twitter activity by team members can help enhance sales. Besides, they have a better chance of getting funding. Fischer and Reuber (2011) have further claimed that Twitter is the most fundamental social media platform used by young entrepreneurs. Users on Twitter can follow their favorite organization depending on their personal views, pursue their interests, and tweet with a limit of 140 words. The word limit makes Twitter a fast mode of putting information as people can post many tweets on any given day. A firm can use this service to polish and publish its brand and gain potential customers (Kietzmann et al., 2011). Furthermore, company recruiters can also use Twitter to find the right people to assimilate into the company (Fischer and Reuber, 2011).

Moreover, start-ups also use a social media platform to interact with other members of the start-up ecosystem for mutual benefit (Raza et al., 2020). Start-ups are increasingly using social media to discuss issues, share information, and collaborate with players in a start-up ecosystem. Social media networking facilitates start-ups in accessing tangible resources such as capital and labor and intangible resources, such as social support, information, and reputation (Bates, 1997; Westhead et al., 2004). Besides, through social networking, start-ups can develop new creative business ideas to enhance their performance (Witt et al., 2008). This networking is also beneficial to start-ups by alleviating ambiguity and edifying the decision process (Autio et al., 2013). Networking facilitates start-ups in reaching out to a venture capitalist (VC) firm. Investors are interested in financing a start-up proposal suggested through their networks. Intangible resources, such as knowledge and experience, are crucial factors that contribute to the success of a start-up (Roure and Maidique, 1986). Here, social media has a role to play by enhancing the effectiveness of knowledge management by substantially enhancing coordination and important knowledge map among team members (Ali et al., 2019). Pitafi et al. (2018) observed that social media adoption facilitates an employee in innovational work and knowledge-sharing relationships. However, personality influences the self-efficacy and online collaboration of an individual (Zheng et al., 2020). Also, social media addiction affects individual performances because of work-technology differences and self-esteem (Khan et al., 2021).

Besides, to have sustainable growth, start-ups must reach out to a maximum number of people. Social media is an effective tool in the current era compared with traditional tools to reach the masses. However, to get the maximum reach out, start-ups must have to ensure their presence in social media sites where they have a maximum number of prospective customers who are hanging out (Sharma and Bharathi, 2013). That is why social media presence is becoming imperative for corporations and start-up firms (Kaplan and Haenlein, 2010). Social media platforms have restructured business management and strategic thinking; and, subsequently, they have introduced a new form of start-up to start-up and start-up to ecosystem communications (Kietzmann et al., 2011). As a result, social media has been hailed as an asset for nascent novice entrepreneurs suspicious of entering a market (Fischer and Reuber, 2014).

Moreover, this helps mitigate the risk attached to start-up firms (Le et al., 2019). Besides, start-ups find social media enabling start-ups, reaching out to prospective customers during crises (Gruber et al., 2015). Likewise, in the early stage of the life cycle, start-up teams have to work in a resource-constrained situation. Working in a hazardous environment, arduous scheduling tasks, and working with multiple teams create stress on team members (Nawaz Khan et al., 2020). However, evidence has shown that social media presence has improved the crisis management skills of teams (Alexander, 2014), which enhances the sustainability of a start-up. The complex and competitive world of business requires that entrepreneurs ensure their presence on social media to communicate with their stakeholders in general and customers in particular (Gratell and Dahlin, 2018). Networking with the players in a start-up ecosystem is a prerequisite to start-up sustainable growth. Thus, social media is crucial for entrepreneurs of today, as it can yield exceptional results if an entrepreneur understands the ecosystem (Bashar et al., 2012). It is pointed out in the literature that the need for social media to do effective entrepreneurial communication is scarce; however, this subject has not been well-researched. Therefore, the focus of this study is on social media adoption on the sustainable growth of start-ups.

### Methodology

#### Research Strategy

The study is conducted to investigate social media adoption on start-up sustainability. This is a qualitative study, where we have applied an inductive research strategy. The case study methodology was adopted to determine the role of social media in sustainable start-up progression. The case study approach is best suited when the goal of a study is to examine a complex subject in detail. Case studies methodology is practical when the purpose is an in-depth analysis of a situation or problem. As a result, one will be better positioned to understand the issue (Noor, 2008).

Thus, quantitative research aims to test, predict, control, and verify phenomena, while qualitative research is focused on human experiences (Creswell and Poth, 2017). Qualitative research is centered on capturing the complexity of human experience through an inductive process, collecting data where participants experience their problems and through an interpretive approach (Creswell, 2007). Inductive research believes that the themes and dimensions emerge from data (Bryman and Becker, 2012). Qualitative research focuses on a bottom-up approach, i.e., the inductive approach (organizing data and themes) means results emerge from data themselves (Creswell, 2007). A semi-structured interview with open-ended questions is used to collect data. An interview is defined as a verbal or oral discussion where an interviewer endeavors to obtain information and get knowledge from an interviewee (Rowley, 2012). The semi-structured interview allows the researcher to explore the experience of participants and gain insight into the issue under study.

The purposive sampling technique is used in this study to select respondents, and it is an effective and appropriate sampling technique for qualitative studies, particularly in research adopting case study methods (Palys, 2008). In the study, we interviewed five people; the first participant is working in a start-up as a brand and social media manager, the second participant as an entrepreneur, and the last three participants are digital marketers and content writers. A small sample size can be sufficient and accurate information within cultural context if participants have a certain degree of expertise in the domain of inquiry (Romney et al., 1986).

The prime objective is to explore the role of social media factors in start-up sustainability, while the secondary goal is to identify the weaknesses in the current social media policies adopted by start-ups. The findings would facilitate a start-up in better organizing its social media strategies.

The interview of five individuals from social media and start-up community, the detail of which is already mentioned, is used to collect data. In the case study, a research interview is the best technique to gather data. However, due to COVID-19, online interviews are conducted through the mutually agreed online meeting portals. These interviews were audio-recorded with the consent of the participants. Each recorded interview was transcribed for data analysis.

## Findings

The study explored the role of social media, an element of a start-up ecosystem, in start-up sustainability. The start-up life cycle model suggested by Maurya (2012) has been adopted to conduct this study. According to the model, start-ups pass through three phases during their life cycle and development, the problem/solution fit stage, the product/ market fit stage, and the scale phase. The respondents of the study are a blend of people from start-up and digital marketing domains. As already mentioned in the methodology chapter, data collection is done through interviews. First, we asked the respondents about the role of social media in start-up sustainability. In the view of respondent #1 usage of social media handles by start-ups creates visibility and talkability with customers and other ecosystem players. It helps them stay in the game as social media keeps them in touch with their customers. They are, thus, improving the chances of start-up sustainability. Since it is a very rocky journey, it is not a smooth ride from a start-up to a mature company. Besides, social media handles are effective tools to announce any changes in your organization or the product offering as per the way you wanted to rather than people grasping inaccurate rumors from the market that might hamper investor confidence besides safeguards against customer retraction. Respondent #2 replied that statistics show that 8 out of 10 people are now using social media. Moreover, it is free as well as people are aware of this tool. Thus, the influence of social media is increasing tremendously; therefore, start-ups can utilize social media to reach out to their current and prospective customers and other players of start-ups. Here, the respondent gives an example, start-ups like Daraz and Foodpanda made their social media influence side-by-side with their apps; this helps gain market recognition, so, in the respondent's opinion, there is no more versatile tool in existence than social media. Respondent #3 considers that the most prominent role of media platforms in creating sustainable start-ups is connecting them with the targeted market. These platforms are a tool to reach customers at the lowest possible cost, and they are bridging the gap between a buyer and a seller where distance is insignificant. In this regard, respondent #4 acknowledged that the role of social media is vital in the dynamic business world. Therefore, one needs to be smart, productive, and well-communicated about business functionality. Besides, you give knowledge to the customer about your product or services should be parallel to your conductivity, which means what you promise must deliver or delight (wow! factor) your client/customer. Respondent #5 believes that social media adoption can boost a start-up by capturing the target audience through attractive, catchy promotional ads for brand awareness. Hence, social media acts as a game-changer for them.

Then, we sought the views of the participants about how social media can facilitate a start-up throughout its life cycle. There is consensus among the participants that social media has a role to play during the start-up life cycle. Respondent #3 believes that social media platforms can facilitate a start-up throughout its life cycle. In the earlier stage, social media platforms help in opportunity identification. In contrast, in the product development stage, promotions are announced *via* social media. Finally, in the maturity phase of a start-up, it provides feedback from both satisfied and dissatisfied customers. Moreover, a social media platform offers an opportunity for a start-up to satisfy a disgruntled customer while it converts satisfied customers into an influencer. Respondent #2 considers that social media has a significant role in brand awareness, thereby attracting potential customers. Moreover, this is the cheapest way to reach the masses and later filter them according to a particular category. Besides being a digital marketer, the respondent firmly believes that an effective social media marketing campaign would prolong the life cycle of a product or service during the maturity and decline stages. Respondent #5 experienced that if start-ups appropriately utilize social media marketing (SMM) tactics, influencers, or paid marketing, they have rightly created awareness of the product/services and a footprint of the product in the brand of customer. Respondent #4 has emphasized social media adoption from the early days of start-up. It would benefit a start-up when taxing on the runway for take-off. Since people recognize you are a start-up, they are ready to avail of a service or product from you; while in the decline stage, social campaigns are a source of product repositioning. Similarly, respondent #1 emphasizes that the launch stage is more about growth hacking; thus, it requires creating awareness. Most start-ups work on a cumbersome burn model that attracts new customers and new business partners through a behavioral change. That is why in the initial stages, start-ups announce unique product offerings and value-added services to mass consumers and potential customers. While start-up grows at a rapid pace and once reaches maturity, it is more about building a brand, and that is where the social media platforms come in very handy. During this phase, they announce campaigns to social causes that they care about or any value-added service they offer to customers, so all this is about brand-building for the maturity stage. In the earlier stage, cash-starving start-ups cannot afford call centers for complaint resolution, so social media is an excellent cheap alternate in such circumstances.

Regarding the third question about how they look at social media as a saver for failing start-ups, this enhances their survivability. Respondent #3 has observed that an influencer plays a vital role in reviving a failing brand. Since social media is also a significant influencer, there is a chance a properly articulated social media campaign can do the miracle for a nearly failed start-up, so in his opinion, failing start-ups should take influencers on board. Here, affiliate marketing is one of many techniques that could help start-ups re-establish themselves. Likewise, influencers could be previously satisfied customers. While respondent #2 has shared a case of the coffee brand Waghera, it has outlets in different parts of the country. According to the owner, after 1.5 years of operations, they did not have enough money to pay the salaries. Suffice it to say, it was a failing establishment, but then they grew and the secret to that growth was social media marketing. They gave people brand awareness and ran campaigns on social media. The story ensures that you are open to suggestions of the people; it helps the owner understand the wants of customers and trends to boost sales. Similarly, suppose start-ups are having a hard time selling their offerings. In that case, it can use social media marketing by making catchy targeted ads, so the consumers become curious and come in to buy the service and judge the hype. Moreover, we also see that on social media, we see one trend which becomes a hit, and then every other brand rushes to take a bite of the pie. For example, the wide-scale adoption of “memes,” which are just funny pictures on the Internet, to market a product has also sky-rocketed. However, previously, they were considered informal, and companies stayed far away from them. Here, respondent #5 has shared a case of a service provider start-up that has faced customer iteration during the early days of the COVID-19 pandemic. The start-up has chalked out an excellent social media marketing campaign to reach out to the clientele during the lockdown. In a short time, the start-up was able to generate enough cash flow. The respondent further added that the right strategy would work and change the losing firm into a profit. Respondent #4 has said that social media is a tool to communicate with a broader audience; thus, one can use it in a way one wants, so it may not give you hyper-growth, but it will create a level of dependability and trust with your customer and the community you operate in, which will subsequently help you on rainy days. Respondent #1 added that the start-up industry is very vibrant the world over, moreover, in the MENA region, Pakistan, India, United Arab Emirates, Egypt, and Saudi. That is why start-up releases the most dynamic and aggressive content on social media; at times, such content gives you great reach, visibility. so, most of the time, you will see social media handles of different start-up companies trending number 1 on Twitter in different markets; this is because of the creativity of the content they share. They creatively touch sensitive issues of society and try to spread a positive message, but sometimes these efforts backfire. However, either way, you get many reaches, and visibility and that at the end of the day, from a marketing and business standpoint, helps you grow, so, whether it is negative or positive (which is even better), you get much reach in society, so these companies release such vibrant and aggressive campaigns in the market. You know this is one way to turn tables around at tricky corners, so this is one way in a short period to improve your growth numbers and business numbers. Although it might not be a sustainable approach, it can give you the type of reach you want on social media channels once a season, so this is one way to save failing start-ups by utilizing their social media and releasing vibrant and aggressive campaigns and content with the community of customers to help you regain momentum in your numbers.

Lastly, we are interested in knowing the appropriate social media strategy for start-up sustainable growth. Here respondent #4 has argued that social media moves fast, while start-ups also go through a period of changes. Therefore, start-up social marketing strategies should be lively and should be reviewed and adjusted as needed. Respondent #2 has revealed many social media strategies that various businesses are currently employing. With the sheer power of social media, any type of strategy can yield exceptional results if well used. Start-ups can use unpaid marketing; there are pages and public Facebook groups. On these groups, freelancers and start-ups advertise themselves by posting their products or get guidance from industry-leading experts. This non-paid marketing also includes the use of “memes,” as mentioned earlier. In comparison, paid marketing through social media is a revolutionary strategy. It is more efficient and cheaper than TV advertising because you would have to hire a team, and you will have to hire actors, which is costly for a young start-up. Industry giants, such as Samsung and Huawei, have also adopted this strategy. We use social media more than we consume digital media nowadays. That is why it has a more significant impact on the mind of a consumer. As a result, it makes a brand more recognizable and increases sale volume. Respondent #3 has suggested that start-ups should hire a social media company. Since start-up teams may be experts in product development, they do not know how to manage social media platforms and get the most benefit from social media platforms as they are just beginners. The purpose of hiring is to understand the industry, understand the market, understand the customers, and then step two to share your KPI's. You should share your short-term and long-term goals with that social media management company. It will help the start-up create a strategic social media policy that will benefit them in the short and long run. Respondent #5 suggested that social media marketing strategies such as wisely optimizing social media pages, using influencer marketing, and having live sessions are critical social media tools that start-ups can utilize for their sustainable growth. According to respondent #1, the most important thing is to have a strategy. Unfortunately, the way the respondent has seen start-ups work in this part of the world, especially the communication they release on social media, does not seem to be thought through, which should not be the case, and thus it is crucial to have a year-long strategy. The first thing to do is to make a calendar for the different events in your demographic. For instance, starting the year, we get the 14th of February, Valentine's Day, an excellent opportunity to cash in on for the market to make an incredible campaign on valentine. Similarly, there are national, religious, and social events in every country, so you have to plan all year round to keep engaging with your customers, which the respondent believes is the only sustainable way to utilize your social media for growth and the best of your business, but unfortunately, that does not happen. So, first, you need to have a clear view of what your purpose is. Then, it is essential to create a strategy to build a brand over the long term. These aggressive campaigns or these sorts of one-off stunts can get you to reach for a certain amount of time. However, for a longer time, you need to be continually engaging with your customers. At times this community of customers and stakeholders will reach out to you in your inbox, in your comments, with all the problems and the nasty stuff they have to say about your company. However, you know, as a tech start-up and as a business that is trying to disrupt the social-economic system, this is something that you should be prepared for. It is crucial to have a strategy. It is imperative to have a year-long strategy aligned with your purpose and communicate with society on different happenings. That, the respondent believes, is something start-ups should look into and plan accordingly as well. It takes time to build the best brand, but you should have a mature plan to achieve that goal. Thus, it is essential to have a clear strategy for the 365 days of the year to build a brand accordingly.

## Discussion

In the dynamic world of business, the role of social media is exponentially increasing and becoming important; start-ups are no exception. Social media tools create significant opportunities for individuals and primarily for entrepreneurs (Fischer and Reuber, 2011). Thus, the development of digital technology in the entrepreneurial sphere is a challenge for both policymakers and entrepreneurs (Thomas et al., 2019). It has necessitated the need to have a clear policy for social media portals. Since it can create visibility for them, please stay in the game with start-ups having in their early years. To achieve this end, start-ups should adopt social media right from their inception. Moreover, being the cheapest mode of reaching prospective customers for cash-starving start-ups, it helps start-ups in creating a wow! factor to their client/customer. Social media enables enterprises to showcase their offerings and what customers value (Sharma and Bharathi, 2013). Social media is a valuable tool to the public the strategic changes being brought about, or policy being implemented, hence deterring against rumors being spread in the market, shattering investor confidence or customer retraction. Social media as an influencer tool helps entrepreneurs persuade their earlier partners (Fischer and Reuber, 2014). Thus, social media plays an important in start-up sustainability.

Social media platforms are the cheapest way to reach the masses. An enterprise can use social media platforms to communicate with its customers and gain trust (Kietzmann et al., 2011). Its role for a start-up changes with the changes in the lifecycle stage. Adopting social media at the nascent stage of a start-up is a tool to create awareness and create a start-up image and recognition in public; it would help them grow. Besides, social media is a vital source to make public the offerings of a start-up, moreover, a medium to listen to dissatisfied customers, resolve their issues/problems, and bring them back, since affording a call center is not possible for cash-starving start-ups. Social media enhances the organizational capability to collaborate, communicate with stakeholders, build customer relationships, and manage knowledge (Smits and Mogos, 2013). Since start-ups multiply, it is time to create a brand here, and social media plays a catalytic role in achieving this goal. While recruiting new customers is quite expensive at the decline stage, converting satisfied customers into influencers will prolong the maturity stage.

Social media is a crucial influencer since it is followed by many. Therefore, failing start-ups, by utilizing their social media and releasing vibrant and aggressive campaigns and content with the community of customers, will regain momentum in your numbers. Besides, catchy advertising to a targeted audience through social media can also be a good option. Moreover, start-ups can utilize this media as an affiliate marketing influencer by hiring satisfied customers. In short social media can provide much-needed reach to failing start-ups, thereby improving their numbers and business (Ghezzi et al., 2016; Basri and Siam, 2017).

Start-ups are always looking for sustainable growth. To achieve this goal, they must make a comprehensive social media strategy to operate in the dynamic environment in the context of technology, markets, and competition to have sustainability (Nambisan, 2017). One suggestion is to hire social media companies to benefit from social media platforms since start-up teams are not aware of this medium. Still, an imperative recommendation is to have a long-term strategy aligned with the goals of the start-up, an aspect that is missing in the start-ups of this region. To have long-term sustainable growth, start-ups should develop a comprehensive social media strategy. Then, they should make a year-long plan to engage their customers; it is the only sustainable way to utilize your social media for long-term sustainable growth.

## Conclusion

The goal of the study is to explore the role of social media adoption in the sustainable growth of start-ups and current social media practices in the start-up sector. The study followed a qualitative case study approach to achieve this objective. The overarching focus of this study was on themes, namely, social media adoption influencing start-up sustainability during various stages of start-ups, survivability of a failing start-up, and, finally, sustainable growth of a start-up. The findings reveal that social media can be of great benefit to start-ups throughout their life cycle. It can be a source of communication with customers and for complaint resolution. Besides, it is the cheapest source of marketing to the masses and segments of society.

Moreover, if they adopt the appropriate strategies, failing start-ups have a good chance of revival. We conclude that social media can play 2-fold instrumental roles. First, it is one of the essential sources of marketing of a start-up. It can lead toward sale augmentation and contribute to the brand equity of firms. Second, social media can also contribute to the improvement of business processes. It implies that social media might be utilized to execute business processes besides marketing and promotion activities. The study concludes that social media can be a vital source to reduce the failure rate of start-ups. However, the downside is that start-ups of the region lack articulation of a clear and precise social media policy to gain maximum out of this vibrant tool.

## Implications and Limitations

It is better to take the key players of social media, practitioners, and members of the start-up ecosystem on board to chalk out a comprehensive policy for the sustainable development of a start-up. Start-ups should articulate a long-term strategy as per the nature and requirement of their business for engaging social media. Social content should be created by keeping potential customers and culture in mind. The findings imply that a non-conventional way of using media can accelerate business growth manifold. Embedding social media with a business process can be a sustainable competitive advantage. Thus, an appropriate digital strategy is required to infuse social media in business processes.

This study has viewed social media adoption in general. In future studies on each social media platform, there is a need to separately examine the impact of each social media platform on start-up survivability. Another limitation of this study is that it investigates the start-up sector only, so in the future, a similar study could be conducted for other sectors to explore the role of social media in their progression. Besides, this study explores start-up survivability by social media adoption. However, start-up survivability might be probed by the adoption of other online tools, such as websites.

## Data Availability Statement

The datasets presented in this article are not readily available because it is a qualitative survey. The respondents have requested to not share their identity. Requests to access the datasets should be directed to saeed@jinnah.edu.

## Ethics Statement

The studies involving human participants were reviewed and approved by the Research Ethics Committee of Mohammad Ali Jinnah University, Karachi. Written informed consent to participate in this study was provided by the participants.

## Voluntary Participation

Moreover, there are tremendous challenges in qualitative research related to privacy concerns of a participant, lack of consent, deception, and participant security concerns, because of research complexities. In this respect, it is worth mentioning that all the participants of the study were recruited voluntarily. Their consent to participate in the study was sought before scheduling an interview. Furthermore, they were properly briefed about the purpose and aim of the research, and the rationale for recruiting them as participants in the study. After that, it is their decision whether they want to participate in the study or not. Moreover, a consent form was sent to them to sign and return to the researcher. Once we received the signed consent form, then we scheduled their interview session.

## Author Contributions

MMuj conceived the idea and helped in writing the introduction and literature review. He has also interviewed the participants, analyzed the interviews, and wrote the discussion part. MMub wrote the conclusion and implications of the study. He also helped in writing the Literature Review. All authors have read and approved thefinal manuscript.

## Conflict of Interest

The authors declare that the research was conducted in the absence of any commercial or financial relationships that could be construed as a potential conflict of interest.
